# Influence of Skin Phototype on the Level of Pain Perceived by Patients Receiving Enoxaparin: A Cross-Sectional Study

**DOI:** 10.3390/nursrep12040092

**Published:** 2022-12-02

**Authors:** Candelaria de la Merced Díaz-González, Josefa María Ramal-López, Juan José González-Henríquez, Milagros de la Rosa-Hormiga

**Affiliations:** 1 Department of Nursing, Faculty of Health Sciences, University of Las Palmas de Gran Canaria, Juan de Quesada, 30, 35001 Las Palmas de Gran Canaria, Spain; 2 Department of Mathematics, Faculty of Health Sciences, University of Las Palmas de Gran Canaria, Juan de Quesada, 30, 35001 Las Palmas de Gran Canaria, Spain

**Keywords:** skin, phototype, pain, low molecular weight heparin

## Abstract

Low Molecular Weight Heparin (LMWH) is commonly used as an antithrombotic in patients with reduced mobility. Its administration is performed by invasive technique (injections) that can cause pain: (1) Background: Pain and bruising are the most common side effects in patients treated with LMWH, but the skin phototype (PT) has never been included; (2) Methods: A cross-sectional descriptive study, developed in the Hospital Unit of Orthopedic and Trauma Surgery over one year. To classify all participants in the sample considering their skin PT and the different pain levels “during” and “after” the administration of enoxaparin. The STROBE checklist was used to evaluate the study. Data analyses were carried out: descriptive statistical analysis and analysis of Variance ANOVA of a non-parametric factor; (3) Results: The sample was 202 participants. The most frequent skin PTs were PT II 43.6% and PT III 33.2%. Mean pain after injection (1.96) was greater than pain during injection (1.4). Better natural protection against sunlight (high PT) would indicate greater post-injection pain; (4) Conclusions: Participants with a medium-high phototype (≥III) perceive a greater pain sensation than participants with a low phototype (≤II) after the administration of enoxaparin.

## 1. Introduction

Enoxaparin is an antithrombotic medication that is administered both intravenously (dialysis circuits) and subcutaneously, the latter being the most common route of administration. Nursing professionals perform this procedure daily in the hospital setting, according to the medical prescription. Localized moderate itching, ecchymosis, hematoma, and pain are among the most common adverse reactions described by AEMPS (Spanish Agency for Medications and Healthcare products) [1]. As regards pain, many researchers have found a very high percentage, where pain may affect up to 94.2% of participants [2]. Many authors have searched for independent variables related to adverse reactions (AR), including the following in their studies: pre-injection cleaning, type of antiseptic, the formation of a skin fold, the length of time to cross the skin barrier, the aspiration, the length of time required by nurses to complete the administration process, the injection of the air bubble, the patient’s age and sex, dose of low molecular weight heparin (LMWH), type of LMWH, and the value of the skin fold [3,4,5,6,7].

A review of some areas of theoretical knowledge related to antithrombotics LMWH prophylaxis (hematology, pharmacology, nursing, and dermatology) helped us find a new inclusion variable for our study in the area of dermatology (photodynamics), skin phototype (PT) [8].

In this sense, it is necessary to continue searching for new independent variables that can influence the occurrence of adverse reactions caused by the administration of enoxaparin, and detect potential risk groups, to guide further research on these variables and help to find a modified injection technique for such group of participants to decrease adverse reactions (pain). This could result in a clinical benefit since it could improve the population’s adherence to treatment.

### Background

Enoxaparin adverse reactions: Enoxaparin is an LMWH, a heterogeneous substance (glycosaminoglycans) obtained from classical or unfractionated heparin through different methods of chemical or enzymatic depolymerization. In this sense, products are structurally different, and have different anticoagulant and antithrombotic capacity.

The Spanish Agency for Medications and Healthcare Products (AEMPS) [1] refers in the technical data sheet of enoxaparin to the following adverse reactions: hematomas at the injection site, edema, bleeding, hypersensitivity, swelling, mass, pain, or reaction (≥0.01–0.1%).

Some authors agree that there are factors inherent to the injection technique itself that lead to the appearance of skin lesions, bruises, and pain [3,4], making proposals [2] to modify the technique, stating that the application of LMWH for 30 s reduces pain and bruising at the injection site. Other researchers [5] propose the elimination of one of the steps in the process of this technique (the skinfold) in the application to obese patients.

Skin and its structure: The skin is a superficial organ, the largest organ in the human body [9], which covers and protects its external surface. The total surface (in an adult) can reach 2 square meters and its thickness reaches 4 mm (mm). It is the largest sensory organ in the human body and is made up of different layers [10]: epidermis, dermis, and hypodermis. Moreover, the skin has a vascular system and nervous nets, which can be both sensory and motor [11]. Therefore, crossing this organ with a needle can cause pain and rupture of blood vessels, producing, in this sense, bruising.

Skin phototypes: Röcken et al. [9] state that variation in skin color is caused by subtle differences in melanogenesis. This is a process that depends on the chemical structure of melanin and melanosomes, although it does not depend on the number of existing melanocytes. Although the skin contains several types of melanin, eumelanin predominates in dark skin. Melanosomes are larger, contain more pigment, and are slower to synthesize. In red-haired individuals, pheomelanin concentration levels are higher than eumelanin levels.

Fitzpatrick in 2009 [12] referred to six skin PTs (see Table 1) considering skin color and the easiness to get sunburned. These phototypes range from type I (the type of skin which is the most sensitive to sunlight) to type VI (the least sensitive skin).

Some research shows that in the Mediterranean and Continental regions of Spain the percentage of skin phototypes that predominate are PT II and III (57.6% vs. 26.1%) [13], as in the Canary Islands (Spain) [14] with PT II 51.5% vs. PT III 37.9%.

Although the skin PT variable has not been included in studies related to LMWH, studies [8] in other lines, such as photodynamic therapy (physical-chemical method), show the existence of a relationship between skin phototype and pain, presenting patients with low skin PT greater pain than those with high skin PT.

IAM: (1) To classify all participants in the sample considering their skin PT and the different pain levels. (2) To identify the relation between the PT of the patient and his/her perception of pain “during” and “after” the administration of enoxaparin.

## 2. Materials and Methods

We followed the Strengthening the Reporting of Observational Studies in Epidemiology (STROBE) [15] recommendations in this study.

### 2.1. Design and Context

It is a cross-sectional descriptive study.

### 2.2. Setting

Its study was developed in the Hospital Unit of Orthopedic and Trauma Surgery (HUOTS) at the Hospital Insular de Gran Canaria (HIGC) over one year, allowing the capture of participants and evaluation.

### 2.3. Participants

The participants in the study have been obtained from the patients admitted to the HUOTS during the study period. The inclusion criteria were over 18 years of age, on anticoagulant treatment with LMWH (Clexane^®^), absence of alterations at their level of knowledge, and written consent to participate in the study.

The selection process was carried out: (1) reviewing daily the new admissions in the unit (from the previous day); (2) Accessing the Electronic Medical Records (EMR); (3) Verify that you do not have any type of diagnosed cognitive impairment or temporary disorientation; (4) Go to the treatment prescribed by your traumatologist, verifying the presence of LMWH; (5) Go to the patient’s room, present the information sheet, data protection, and informed consent; (6) Acceptance by the patient allowed inclusion in the sample of participants.

### 2.4. Variables

The variables included in this study are found in Table 2; sociodemographic, independent, and dependent variables.

### 2.5. Data Sources and Measurement

The procedure that is always carried out by the HUOTS nurses is as follows: (1) to check the prescription; (2) To inform the patient; (3) To provide the patient with a quiet and private environment; (4) To select the injection site; (5) To clean the area and to form the skinfold; (6) To insert the needle forming a 90-degree angle with the skin surface. This should be done relatively quickly without moving the needle; (7) To keep the skin fold tight all the time until the removal of the needle, (8) To inject the medication (variable period); (9) To remove the needle quickly and in the same direction without moving it; (10) To leave the cotton or gauze if there is blood loss while applying slight pressure; (11) To explain the importance of avoiding massaging the area; (12) To record the medication administration in the patient’s EMR.

The evaluation tools used for data collection were: (1) REM (provides the researcher with information on compliance with the inclusion criteria and provides data on the variables included in the study: sex, age, among others); (2) Stopwatch (measure injection time and 2 min after finishing it); (3) VAS pain scale (measure pain during and 2 min after injection); (4) PT Skin Scale (Fitzpatrick) [12] (evaluate skin type before sun exposure).

Measurement procedure: (1) The researcher witnesses how the one of the five nurse applies the injection and times the time taken (<10 s or >10 s) and the timer is activated again; (2) The participant is asked by researcher about his perception of pain during; (3) After 2 min of completion of the injection, the participant is asked about the pain afterward; (4) To identify the participant’s PT (Fitzpatrick scale—Table 1) [12], and he participant is asked by reaseacher about his/her skin condition during regular sun exposure.

### 2.6. Bias

Among the biases that have been able to influence the measurement of the level of pain, is the fixed prescribed dose of analgesia. Because this prescription could not be avoided, in the participants who were administered analgesia at 4 p.m., enoxaparin was administered as late as possible at 8 p.m.

### 2.7. Study Size

The minimum sample size was 167 participants, based on a 50% response distribution a 5% margin of error, a 95% confidence interval, and a 7% permissible error, considering a mean of annual admissions in the HUOTS 1113 patients

Inclusion and exclusion criteria: to select the participants in this study, the following inclusion criteria were established: patients hospitalized in the HUOTS of the HIGC, over 18 years of age, on anticoagulant treatment with LMWH (Clexane^®^), absence of alterations at their level of knowledge and written consent to participate in the study.

### 2.8. Quantitative Variables

The estimation method and the following statistical methods were used: to present the data, the method of descriptive statistics was used—arithmetic mean (M), the value of which determines the average level of a given variable, and standard deviation (SD), a statistical measure of scattering the results around the expected value.

### 2.9. Statistical Methods

After data collection and measurements were carried out, they were registered in a database created for this purpose, which was statistically analyzed with the statistical software Statistical Package for the Social Sciences (SPSS) (IMB, Armonk, NY, USA), with appropriate licensing, and R-Project version 3.1.0 (R Development Core Team, Vienna, Austria), which is free software. The following statistical analyses were carried out: descriptive statistical analysis and analysis of Variance ANOVA of a non-parametric factor.

## 3. Results

### 3.1. Sample Characteristics

The sample obtained was made up of 202 participants, with a mean age of 64.39, a range of (32–89), and a Standard Deviation (SD) of 15.04. The distribution of the sample as regards gender was the following: 62.4% (*n* = 126) female participants and 37.6% (*n* = 76) male participants.

### 3.2. Subject’s Skin PT and His/Her Perception of Painful Sensation Perceived by Enoxaparin Recipients

The skin PT or sun sensitivity was distributed considering the following classification: PT I 10.4% (*n* = 21), PT II 43.6% (*n* = 88), PT III 33.2% (*n* = 67), PT IV 12.9% (*n* = 26) and PT V and VI 0% (*n* = 0).

The mean for painful sensation perceived during the administration of the LMWH injection in the study sample (202 participants) increased to 1.4 with a range of (0–6) and a SD of 1.35, whereas the mean for painful sensation perceived after the administration of the injection was 1.92 (0–8) with a SD of 2.08. To determine the levels of pain perceived by participants, the pain was represented in Table 3 (absolute frequency and percentage) considering the skin PT. As regards pain during the administration of enoxaparin, it can be observed that the highest percentage of participants in the sample present levels of pain which range from 0 to 2 in all PT, whereas the sample is more evenly distributed among the different levels of pain concerning pain after the administration of enoxaparin.

The means of pain during and after the administration of this medication as regards PT are shown in Table 4, both the mean in the total participant group in our sample and the mean in those participants who presented skin injuries (*n* = 171). This table shows again the progressive increase of the mean in pain during the administration of enoxaparin as the skin PT increases in the total sample. As regards pain after the administration of enoxaparin, the pain increases, concerning PT, to the level of PT III, to subsequently decrease, keeping a high mean as regards the lowest PT.

Additionally, Table 5 shows the relation among different categorical variables. Among these relations, we must mention that established among PT are the variables “pain during the administration of the injection” and “pain after the administration minus the pain during”. Since the variable “post-injection pain” is moderately related to pain during the administrations of LMWH, we have analyzed the difference between these two variables to assess a possible increase or decrease of post-injection pain.

As regards PT (Table 5) we can observe that post-injection pain increases with the value of PT as compared to pain during the administration of the injection. Thus, better natural protection from sunlight (High PT) would indicate a higher post-injection pain. Nevertheless, as regards pain during the administration of enoxaparin, PT does not seem to have any relation with the modification of pain perception.

## 4. Discussion

Sample characteristics: the mean age sample was 64.39. It must be considered that 66.8% of the sample is made up of individuals over 60 years old. This fact reveals that the increase of the mean age, due to the increase of the elderly population in the world, is associated with the increase of Venous Thromboembolism, as stated by many authors [13].

A total of 62.4% of the sample is made up of female participants, as is shown by other researchers [2,4,16,17]. This fact coincides with the gender distribution in the Spanish population in 2021 [18], where the percentage of women is higher than that of men.

The enoxaparin administration procedure performed by HUOTS nursing professionals during this investigation was the type usually performed in daily care practice in this unit.

It has not been possible to compare the results obtained in the present study, where the PT is related to the enoxaparin administration procedure, with previous investigations. After carrying out bibliographic searches that included (PT AND pain) AND (LMWH OR enoxaparin), no results were found.

It is of crucial importance to consider the PT variable in future research on the administration of the different low molecular weight heparins available on the market and the levels of pain these heparins can cause.

The skin PT or sun sensitivity in our sample was mainly type II in 43.6% of participants and type III in 33.2%. Researchers [14] performed in the Canary Islands population showed that the most frequent PT in the population studied was type II (51.5%), followed by type III (37.9%), highlighting the presence of PT II in more than 55% of female participants.

As regards the level of pain during the administration of enoxaparin, 72.4% of the sample presented pain, increasing the mean intensity to 1.4. It should be emphasized that most participants in the sample reported mild pain 1–2 (64.4%), whereas 29.7% had no pain. Nevertheless, some researches have similar characteristics to those in the present study (pre-filled syringes, enoxaparin, the abdomen as injection site, the formation of a skin fold, the insertion of the needle forming a 90-degree angle with the skin surface, no aspiration during the administration, the injection of the medicament in about 10 s, the injection of air bubble, the removal of the needle, the release of the skin fold, and the practice of avoiding massaging the site after the injection), in percentages of pain at “moderate-severe” levels, between 45.5–76.7% [2,6]. Kuzu and Ucar [2] found pain in 94.2% (group I without applying local ice), prevailing mild pain (45.5%), and no pain in 5.8% of participants. Zaybak et al. [19], in their quasi-experimental study, found a mean of 2.06 + 2.23 with the standard technique. Chan H. [4], in his quasi-experimental research with Dalteparin, obtained a higher mean (2.28), without referring to the distribution at different pain levels. In the study carried out by Avşar and Kaşikçi [6], the control technique applied to group II indicates pain in nearly 99% of participants, showing moderate-severe pain in 76.7% and only mild pain in 22.1%. As it has been observed, the percentage of pain and its mean in the different studies performed is highly variable, although the same technique is applied and sometimes with different LMWH. This variable was thought to be the variable that modified pain. Nevertheless, it remains unclear. As regards Dalteparin, low molecular weight heparin salified with calcium, some researchers [20], state that it can cause less pain than that caused by LMWH which are salified with sodium (enoxaparin). However, if we compare this study with theresearch conducted by Chan [4], we can observe that its mean is higher. In this sense, this assumption is not fulfilled. On the other hand, the increase of injection time (30 s) revealed a decrease of pain during the administration of the injection [4,19,21], showing inall the results obtained a decrease of the mean of pain. The interest in the injection site is highly relevant. Although Pourghaznein et al. [7], does not provide the mean age score for pain among their results, they nevertheless claim to have found significantly more intense pain perception when the drug is injected subcutaneously into the thigh rather than administered into the abdominal area.

The present study has shown a low mean pain level as compared to that shown by those studies mentioned above, without increasing the injection time (duration). Nevertheless, it must be considered that the research mentioned above was conducted in different countries, all of them with different cultures and religions. In this sense, pain responses can be conditioned by these factors [22]. Furthermore, we must consider that pain is a subjective experience due to the complex mechanisms of nociceptive processing, which is perceived as a multidimensional sensory and affective experience [23]. This fact leads us to state the importance of incorporating sociocultural variables into future research.

The mean pain score after the administration of the injection was 1.92, which was above the mean pain score during the administration of enoxaparin. The pain was present in 65.8% of participants. 43.1% reported mild pain and 34.2% of participants had no pain. We can point out that these data differ if compared to those data obtained by other authors, since in some cases, the measurement is not performed 2 min after the administration of the injection, or, although the duration of pain is measured, they do not offer the measurement of the level of pain. Therefore, we will try to search for the most relevant data: Kuzu and Ucar [2] found in their study that pain after the administration of enoxaparin (in group I where ice was not used before and after the injection) was distributed as follows: 36.5% of the sample did not present pain, in 35.3% of participants pain lasted more than 120 s, and in the remaining group it lasted between 1 and 120 s. According to this distribution, we can state that 64.7% of participants experienced pain after the administration of the injection, without considering the duration of the injection. These data coincide with those data obtained in our study; the absence of pain was 34.2% vs. 36.5%. Mohammady et al. [24] emphasize that a slow injection may slightly reduce pain 48 h later, while authors [25] affirm that the duration of the injection of 30 s compared to 10 s, produces less pain.

Although it is not a variable included in the present study, however, they make contributions regarding situations where the treatment with LMWH causes pain to the participants. Mohammady and Narges [26], in their meta-analysis, recommend putting cold before or after the injection, 3 to 5 min, which also allows a lower incidence and size of bruises. Similar are the results obtained by Wang et al. [27], where cold was found to be able to reduce pain at 72 h. Additionally, the application of ShotBlocker and cold are approaches that reduce pain and increase participants’ satisfaction with nursing autonomy [28].

Regarding the injection site, the abdomen seems to be the most recommended by various authors [29], as it presents the least degree of pain compared to other areas, such as the arm.

Using a Spearman connection between age with pain “during” and pain “after”, pain “during” was found to be related (0.5; *p* < 0.01) to pain “after”, which could be explained by the intensity and/or duration of “post pain” over “during pain” or by a superimposition of “post pain” over “during pain”.

A progressive increase of the mean for pain during and after the administration of the injection was found, as the PT increased in the total sample. This could be an indicator during and after the administration of the injection if the participant is more likely to have bruising.

A significant correlation between post-injection pain and pain during the administration of the injection in PT III has been observed, as well as higher natural protection from sunlight (high PT) which indicates a higher post-injection pain. Nevertheless, pain during the administration of the injection PT seems not to be the reason for the modification. These results have not been able to be compared since no bibliography includes these study variables (((LMWH) AND pain) AND phototype) (((LMWH) AND phototype) AND pain). However, if we look for other factors that can be applied to the participant and that can measure the pain according to the PT, we find photodynamic therapy (physical-chemical method) [8] where the research concludes that “… patients who are going to experience more pain. Low skin Phototype as compared to high skin Phototype”.

We believe that it is crucially important to clarify that, during the administration of LMWH by the nurses, the researcher evidenced a high cases of skin lesions, such as stretch marks, but this aspect was not evaluated; however, it could be a variable to include in future studies, relating the level of pain with the presence of stretch marks in the injection area. The scarcity of literature on this variable leads us to recommend the inclusion of PT in future research on pain and LMWH.

### Limits 

The captured study sample does not show participants with PT V and VI, so it has not been possible to provide data on pain in these groups.

## 5. Conclusions

Considering the objectives of this research, we reach the following conclusions: (1) The most frequent skin PT in our sample is PT II-III; (2) The variable “phototype” does not influence the painful sensation perceived by participants during the administration of enoxaparin; (3) The skin PT has a significant influence (*p* = 0.01) when that pain perceived by the subject during the administration of the injection is excluded from post-injection pain. Thus, a subject with a low skin phototype is likely to have a lower difference in pain than participants with a high skin phototype; (4) Those participants with medium-high PT (≥III) perceive a higher painful sensation than those participants with low PT (≤II) after the administration of enoxaparin.

## Figures and Tables

**Table 1:** Classification of skin phototype (PT) (FITZPATRICK).

Phototype (PT)	Type of Skin
PT I	The skin always burns, it is never suntanned
PT II	The skin always burns and it sometimes tans
PT III	The skin burns sometimes, it always tans
PT IV	The skin never burns, it always tans
PT V	Moderately pigmented skin
PT VI	Black skin

Table based on: Fitzpatrick TB. Validity and practicality of sun reactive skin types I–VI [12].

**Table 2:** Independent and dependent variables.

Independent Variables	Description
Age	Age of subject in years
Gender	male or female
Dose of LMWH	The prescribed dose of LMWH (enoxaparin—Clexane^®^) is in milligrams. Pre-filled syringe with a single dose of enoxaparin- Clexane^®^, in a single-dose plastic case, consisting of a cap, a plunger, a body, and a 27 G needle that does not allow separation from the body.
Injection time	Time in seconds, from the introduction of the needle in the skin to its removal (withdrawal), options will be: >10 s or <10 s
Skin Type (phototype-PT)	According to the Fitzpatrick scale, the skin phototype (PT) is identified as a function of sun sensitivity. Possible values: PT I (the skin always burns, it is never suntanned), PT II (the skin always burns, it is sometimes suntanned), PT III (It burns sometimes, and it is always suntanned), PT IV (it never burns, it always tans), PT V (moderately pigmented skin) and PT VI (black skin).
Dependent Variables	Description
Pain level during injection	Level of pain that the subject presents “during” the administration of LMWH on a Visual Analogue Scale (VAS) with possible values between 0–10.
Pain level after injection	Level of pain that the subject presents “post” (2 min after) administration of LMWH on a VAS scale with possible values between 0–10.

**Table 3:** Pain levels during and after Enoxaparin injection by Phototype (PT). Absolute frequency (AF) and percentage (%).

		During Injection Pain			Post-Injection Pain			
Pain Level	PT I	PT II	PT III	PT IV	PT I	PT II	PT III	PT IV
	AF (%)	AF (%)	AF (%)	AF (%)	AF (%)	AF (%)	AF (%)	AF (%)
Pain 0	11 (5.4)	23 (11.3)	16 (7.9)	10 (4.9)	17 (8.4)	29 (14.3)	19 (9.4)	4 (1.9)
Pain 1	4 (1.9)	37 (8.3)	17 (8.4)	0	4 (1.9)	24 (11.8)	11 (5.4)	6 2.9)
Pain 2	3 (1.4)	18 (8.9)	26 (12.8)	9 (4.4)	0	4 (1.9)	10 (4.9)	6 (2.9)
Pain 3	3 (1.4)	0	6 (2.9)	3 (1.4)	0	13 (6.4)	6 (2.9)	3 (1.4)
Pain > 3	0	10 (4.9)	2 (0.9)	4 (1.9)	0	18 (8.9)	20 (9.9)	7 (3.4)

* No sample is available PT V–VI.

**Table 4:** Mean pain level during and after Enoxaparin injection by Phototype (PT).

		During Injection Pain			Post-Injection Pain			
Level Pain	PT I	PT II	PT III	PT IV	PT I	PT II	PT III	PT IV
Mean pain level (n 202)	0.90	1.35	1.41	1.96	0.19	1.69	2.58	2.23

No sample is available for PT V–VI.

**Table 5:** Categorical relationship among different variables (ANOVA).

	During Pain				Post-Pain Minus during Pain			
	A ± Sd	Md	*n*	Range	A ± Sd	Md	*n*	Range
Age								
30–39	2.3 ± 1.3 **	2	10	1–4	1.1 ± 0.9	1	10	0–2
40–49	1.2 ± 0.9	1	39	0–2	0.7 ± 1.7	1	39	−2–4
50–59	1.2 ± 0.8	1	18	0–2	0.6 ± 1.7	0	18	−1–3
60–69	1.7 ± 1.8	2	48	0–6	0.3 ± 1.8	0	48	−3–5
70–79	1.6 ± 1.5	1	52	0–5	0.5 ± 1.6	0	52	−2–4
80–89	0.9 ± 0.7	1	35	0–2	0.4 ± 2.1	0	35	−1–6
Gender								
Male	1 ± 0.9 *	1	76	0–2	0.2 ± 1.4	0	76	−2–3
Female	1.6 ± 1.5	1	126	0–6	0.7 ± 1.9	0	126	−3–6
Dose LMWH								
40 mg	1.4 ± 1.4	1	183	0–6	0.5 ± 1.8	0	183	−3–6
60 mg	1 ± 1.1	1	6	0–2	0 ± 0	0	6	0–0
80 mg	2 ± 0.9	2	9	1–3	1 ± 2.3	0	9	−1–4
160 mg	1 ± 0	1	4	1–1	0 ± 0	0	4	0–0
Phototype (PT)								
PT I	0.9 ± 1.1	0	21	0–3	−0.7 ± 1.2 **	0	21	−2–3
PT II	1.4 ± 1.4	1	88	0–5	0.4 ± 1.6	0	88	−3–6
PT III	1.5 ± 1.1	2	67	0–4	1.1 ± 1.9	0	67	
PT IV	1.7 ± 1.9	2	26	0–6	0.5 ± 1.6	0	26	−3–6

* Significant correlation at 0.01 level. ** Significant correlation at 0.001 level.

## Data Availability

Not applicable.
